# Supplementary material for: A digital platform with activity tracking for energy management support in long COVID: a randomised controlled trial
Source: Nat Commun. 2026 Feb 2;17:945. doi: 10.1038/s41467-025-64831-y (PMC12864992; doi:10.1038/s41467-025-64831-y)
Supplement: Supplementary file 1 — Supplementary Information [file 41467_2025_64831_MOESM1_ESM.pdf]

STUDY IMPLEMENTATION

Protocol

Individuals expressing interest were contacted via telephone or video conferencing to arrange a briefing. This meeting included screening for inclusion and exclusion criteria, a verbal overview of the study and an opportunity to ask questions. Participants were provided with an information sheet and re-contacted at least 7 days later when they were provided with a further opportunity to ask questions and then, if willing, were enrolled on the trial. Once enrolled, participants provided their written informed consent to participate in the study and were allocated an enrolment number. A secure third-party service (Study-Randomizer) randomised participants into intervention or control groups stratified by age and sex with recruitment logs to ensure randomisation in recruitment order.

Following randomisation, a study start date approximately one week later was agreed. This provided time for an activity tracker (Fitbit Charge 5) to be posted to those allocated to the intervention group. Subsequently, in an online meeting, a study team member aided participants in downloading the PaceMe app from the relevant app store, ensuring they successfully registered an account and proceeded through the onboarding and digital consent screens. The app then asked for further participant information, including an activity tracker number for those allocated to the intervention group. The tracker number acted as a 'switch' such that participants who did not provide one were taken to the control version of the app, in which the home screen consisted of each of the questionnaires only. For those who did provide a tracker number, their home screen was an enhanced version of the PaceMe app, which included live metrics of their activity pacing for that day and over the previous days (see below).

The intervention used live activity tracking to help people with long COVID to better manage their daily activities so as to reduce the risks of inducing a bout of PEM. To do this, the intervention group had their activity continually tracked using a wrist worn activity tracker (Charge 5, Fitbit, San Francisco, USA). However, the Fitbit phone app does not process activity data in a manner suitable for people at risk of PEM. Consequently, we removed or turned off all of the activity notifications from the Fitbit app and employed our own activity server to link each participant's activity data to their PaceME app. Our activity server accessed the tracker data every 3 minutes and collected the relevant activity data in 1-minute epocs for the current day (heart rate and steps). Once collected, our server processed this data and stored the results in a database that enabled live streaming of each user's data to the relevant part of the PaceME app. This meant each user was able to get personalised, live updates within the PaceME app, giving them an overview of their activity so far for the day along with an indication as to whether this amount of activity had occurred prior to a previous bout of PEM. Participants were free to use this information to better plan their day-to-day activities to avoid over-exertion.

During this process, a member of the research team ensured that all of the alerts, notifications and prompts which encouraged additional exercise, managed by the trackers own app had been switched off or removed. Both intervention and control participants also received automated alerts to remind them when they were due to complete specific questionnaires and reminders if they remained uncompleted a week after the intended completion time. In addition to the instruments, the app also enabled participants to log any issues with the app and register a call back request from the team in the event of any issues.

Activity allowance

Following enrolment, participants were set an initial activity allowance to avoid accumulating more than 30 minutes with a heart rate above 60% of their age-predicted HRmax. This limit was designed to be iterative and revised in response to how well they managed their pacing and whether or not they experienced bouts of PEM. When participants reported PEM, an automatic review of their activity data would be triggered. The review examined their activity data over the preceding three days (since PEM may be delayed by several days). If participants had exceeded their activity allowance in those three days, this would be noted as 'poor pacing', and no changes would be made to the activity allowance. If participants had exceeded their activity allowance in any one of the three previous days, that was considered an 'inappropriate allowance, and the activity allowance was reduced using a stepwise algorithm. Where participants did not log bouts of PEM for three continuous weeks, the activity allowance would be increased again in a stepwise algorithm. It is important to note that during enrolment, care was taken to be clear to participants that the activity allowance was a reference point against which participants could gauge their current activity. It was not intended as a target to be met, and at no point would participants be urged to do additional activity to 'meet their allowance.

In some cases, HR was an inappropriate metric for measuring pacing. Participants may have cardiac autonomic issues resulting in high or unstable resting heart rates, which interfere with the link between current heart rate and activity. Alternatively, participants may experience several recurring bouts of PEM, which, if the activity allowance were continually revised, would result in an allowance too low for participants to feasibly stay within. In these cases, participants were switched to a step-count activity allowance, also using the data from the same tracker.

The initial step-activity-allowance was determined by finding the highest number of steps per day that was not followed by PEM in the following three days. After that, the process of altering the step-activity-allowance was similar. When participants registered PEM, the step-activity limit would be reduced only if they had stayed within their current limit for the previous three days. Where no PEM was registered for three continuous weeks, the step-activity-limit would be revised upwards.

Pace Me App Display

The home screen of the PaceME app included a live display of participants' pacing progress for that day. For those using a heart-rate-based activity allowance, the display showed the heart rate they should stay below, how many minutes they had accumulated above that heart rate, along with an indicator of how much of their activity allowance they had 'used' that day (e.g. if their limit was no more than 30 minutes above 120 bpm, and they had accumulated 15 minutes, the indicator would show 50%;). For those who had been switched to a step count activity allowance, a similar display of the day's step count was displayed instead.

In addition, a separate screen displayed additional information, including a series of indicators. These included a set of emoji indicators showing how well they had paced over the preceding three days to remind them how well they had managed to pace recently. In addition, there were a series of graphs showing (a) when on the current day they had spent most time above their activity allowance, (b) when on average over the last week had they accumulated the most activity (i.e. most active hours of

the day to be aware of) (c) when in the current day they had spent most time resting. The aims of these indicators were to help participants understand the times of day when they were most likely to be overly active and when they tended to get the most rest. In addition, we were cognizant of the risk of overwhelming participants with information, and as such, these additional screens were hidden and revealed when tapping a specific icon.

Messaging

The intervention also provided messages to alert participants as their accumulated time above their activity allowance increased. Our prior PPI work indicated that a concern of participants was receiving too many notifications, which also can reduce participant engagement with an intervention. As a result, our intervention would send a maximum of three pacing notifications per day. In addition, because we had turned off all Fitbit notifications, the PaceME app also took over alerting participants if the tracker battery was low or had not synced with the Fitbit servers for more than 2 hours. As a result, participants could receive between zero and five daily alert notifications.

Pacing alerts were personalised to each participant's current activity allowance, and where interactive notifications were triggered by our activity server when participants had reached 50%, 75% and 100% of their activity allowance. The alert comprised of a standard phone notification with the PaceME logo and a text header '{Name} has reached {xx%} of your activity allowance' (where {Name} and {xx%} represent the participant's first name and the relevant level of their activity allowance that had been reached). When tapped on the PaceMe app opened and displayed an infographic containing a pacing support message. The support messages were provided as part of a separate project to crowd-source pacing advice messages from people with ME/CFS who had used activity pacing for a long time. We curated these responses and collated them into relevant infographics to use as messaging support.

Sync notifications were not interactive, and when tapped they opened the Fitbit app to trigger syncing between the data stored on the tracker and the Fitbit servers. Similarly, the low-battery notifications were not interactive, and a simple notification with a logo and a message that their tracker was battery charge was below 20%.

Data flow for energy management support. A: Participants provided with a tracker and all notifications to increase activity turned off. B: The tracker's own app tracks and stores activity metrics (HR, Steps etc), C: Our server pulls the data from Fitbit every 3 minutes and re-processes to be useful for individualised energy management targets, D: Processed data is streamed to Pace Me app, E: participants log bouts of post-exertional malaise (PEM) which is used to determine the current energy management limit, F: Alerts are sent to the app as participants increase the time spent above their limit.

DATA PIPELINE VISUALISATION

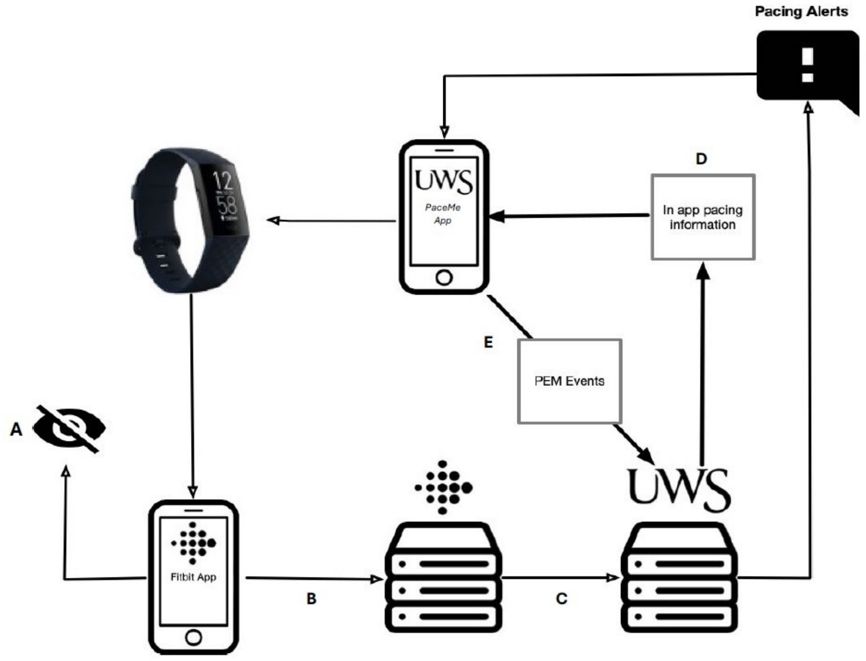

Supplementary figure displaying the data pipeline from the wearable device to the FitBit app, which had notifications hidden from participants (A), then was transferred to the FitBit cloud (B), which we pulled down onto our server (C). This data then updated the participants app with near-live pacing information (D), and then if participants reported PEM events (E), this influenced our pacing algorithm. Finally, the data were used to send pacing alerts when participants reached certain thresholds.

PARTICIPANT DEMOGRAPHICS

Supplementary table displaying participant characteristics of those who completed the study.

	Control (n=77)	Intervention (n=84)
	**Mean (95% CI)**	**Mean (95% CI)**
**Age (years)**	47 (45-50)	45 (43-48)
**Gender (M/F)**	60 F (78%), 17 M (22%)	66 F (79%), 18 M (21%)

EMPLOYMENT STATUS AT BASELINE

Intervention

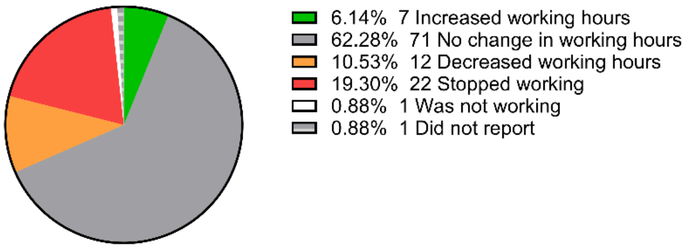

Total=114

Control

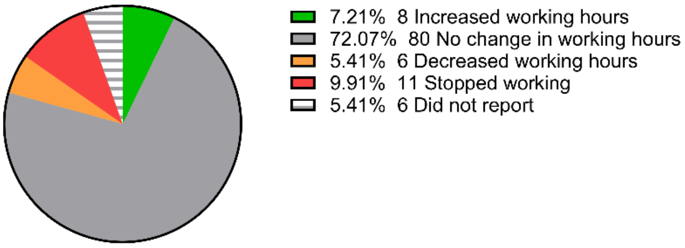

Total=111

Supplementary figure displaying the employment status of all participants at baseline. Not all participants completed the questionnaire, thus why the total n is not 125 per group.

DAYS WITHOUT PEM

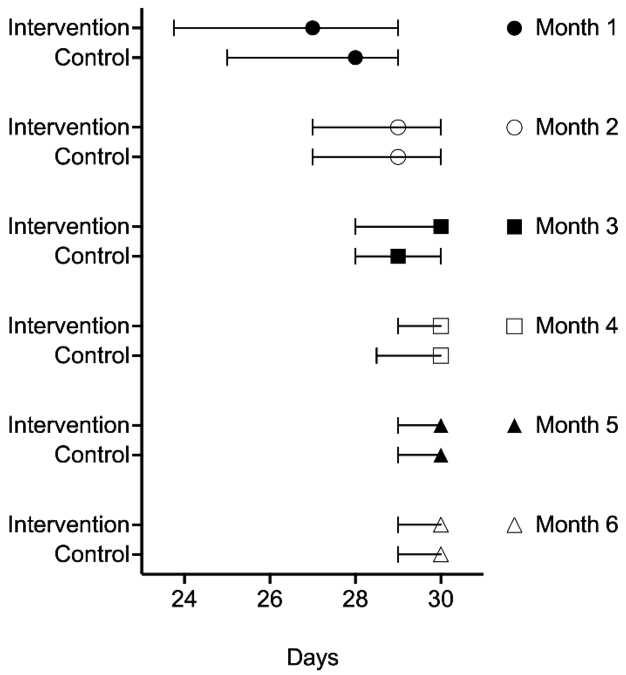

Supplementary figure displaying the mean number of days per month that participants did not report a PEM event. Data are mean and 95%confidence intervals.

STUDY PROTOCOL

USING ACTIVITY TRACKING AND JUST-IN-TIME MESSAGING TO IMPROVE ADAPTIVE PACING: A PRAGMATIC RANDOMISED CONTROL TRIAL

STUDY INVESTIGATOR(S)

Principal Investigator (A): Prof Nicholas Sculthorpe[1]

Email: Nicholas.sculthorpe@uws.ac.uk

Co-Investigator (A): Dr Lawrence Hayes[1]

Co-Investigator (B): Dr Joanne Ingram[1]

Co-Investigator (C): Dr Jacqueline Mair[1]

Co-Investigator (D): Dr Andisheh Bakhshi[1]

Co-Investigator (E): Dr David Carless[1]

1. University of the West of Scotland, Glasgow, UK.

Contents

STUDY INVESTIGATOR(S) 7
BACKGROUND 8
AIM(S) OF STUDY 9
OBJECTIVES 9
HYPOTHESIS 10
STUDY DESIGN 11
STUDY SETTING/LOCATION 11
STUDY POPULATION 11
ELIGIBILITY CRITERIA 12
STUDY OUTCOMES 12
STUDY PROCEDURES 13
STATISTICAL CONSIDERATIONS AND DATA ANALYSIS 19
ETHICAL CONSIDERATIONS 20
OUTCOMES AND SIGNIFICANCE 21
REFERENCES 21

BACKGROUND

People with long COVID report 'push-crash' cycles, with a disproportionate worsening of

symptoms in response to activity, similar to post-exertional malaise (PEM) reported by patients with chronic fatigue syndrome[1–3]. These PEM-like symptoms increase the overall symptom load, reduce each individual's quality of life, and make recovery harder.

Adaptive pacing has emerged as a common strategy to self-manage PEM-like symptoms[4,5]. Our systematic reviews suggest adaptive pacing is effective in improving some symptoms in people with ME/CFS[4,6]. However, recent work endorsed by our long COVID PPI group indicates that implementing adaptive pacing is often problematic for those attempting to self-manage. Difficulties include accurately estimating concepts such as energy availability and predicted energy use and identifying and tracking a suitable threshold to limit activity. Combining these requirements to plan daily activities, often hour by-hour, can be highly challenging, particularly when symptoms include impaired cognition.

This project aims to determine if combining continuous activity tracking with a just-in-time adaptive intervention (JITAI) can address these limitations. JITAIs provide information to participants at a time and context where they can act upon it [7,8]. Widely used in behaviour change research, JITAIs are yet to be applied to adaptive pacing. Using a randomised control study design, we will allocate 250 participants to receive either JITAI supported adaptive pacing or usual care. Our primary outcome is PEM using the De Paul Symptom Questionnaire (DSQ)-PEM [9] at baseline and 6 months.

AIM(S) OF STUDY

This study aims to evaluate the effectiveness of a digital intervention designed to support individuals with long COVID in managing their energy levels and preventing post-exertional malaise (PEM). By integrating a wearable activity tracker with a just-in-time adaptive intervention (JITAI) framework, the study seeks to explore how real-time alerts and retrospective activity analysis can help individuals regulate their daily activity levels. Ultimately, this research aims to determine whether such an intervention can enhance self-management strategies, reduce the frequency and severity of PEM episodes, and improve overall quality of life for people living with long COVID.

OBJECTIVES

- To assess whether real-time activity alerts based on JITAI improve participants' ability to adhere to adaptive pacing strategies.

 To analyse retrospective activity data preceding PEM episodes to refine individual activity thresholds.
 To examine user experiences, feasibility, and adherence to the digital intervention for managing activity levels.
 To determine whether the intervention leads to a reduction in the frequency and severity of PEM episodes over time.

HYPOTHESIS

Primary Hypothesis

H_0: An activity tracking and personalised JITAI will not reduce symptoms of post-exertional malaise (PEM) in people with long COVID compared to usual care six months after randomisation.

H_a: There will be differences in symptoms of PEM in people with long COVID compared to a usual care group six months after randomisation in an activity tracking and personalised JITAI.

Secondary Hypotheses

 Quality of Life

- **H_0**: An activity tracking and personalised JITAI will not improve quality of life in people with long COVID compared to usual care six months after randomisation.
- **H_a**: An activity tracking and personalised JITAI will improve quality of life in people with long COVID compared to usual care six months after randomisation.

 Anxiety and Depression

- **H_0:** An activity tracking and JITAI will not improve anxiety and depression in people with long COVID compared to usual care six months after randomisation.
- **H_a:** An activity tracking and personalised JITAI will improve anxiety and depression in people with long COVID compared to usual care six months after randomisation.

 Breathlessness

- **H_0**: An activity tracking and personalised JITAI will not improve breathlessness in people with long COVID compared to usual care six months after randomisation.

- **H_a:** An activity tracking and JITAI will improve breathlessness in people with long COVID compared to usual care six months after randomisation.

 Cognitive Function

- **H_0**: An activity tracking and JITAI will not improve cognitive function in people with long COVID compared to usual care six months after randomisation.
- **H_a:** An activity tracking and personalised JITAI will improve cognitive function in people with long COVID compared to usual care six months after randomisation.

STUDY DESIGN

This study was designed as a pragmatic randomised controlled trial (RCT) to evaluate the effectiveness of an activity tracking and personalised just-in-time adaptive intervention (JITAI) for managing post-exertional malaise (PEM) in people with long COVID. A pragmatic RCT was chosen to assess the intervention in real-world conditions, with that findings are generalisable to routine clinical and self-management settings. Pragmatic trials consider diverse participant adherence, varied settings, and real-life constraints and this approach was intended to allow for a more comprehensive understanding of the intervention's feasibility, acceptability, and potential for real-world implementation.

STUDY SETTING/LOCATION

The study was conducted remotely within the UK, with participants undergoing the intervention or control conditions in their own homes and during their day-to-day activities. While the study was coordinated from The University of the West of Scotland, all aspects of participant engagement, data collection, and intervention delivery were designed to integrate into participants' everyday lives. This facilitated the evaluation of a scalable digital support platform that could be implemented without requiring in-person contact. Additionally, conducting the study in this way allowed for a meaningful evaluation within the context of a pandemic, where ongoing social distancing measures limited access to traditional healthcare and in-person research participation.

STUDY POPULATION

The study population consists of individuals with long COVID who were not hospitalised during their initial COVID-19 infection and are managing their symptoms independently while

living in the community. To ensure broad representation, recruitment was conducted through support groups and community networks. This approach aimed to reflect the known demographic distribution of long COVID in the general population, considering factors such as the proportion of males and females, age distribution, and the prevalence of comorbidities within the non-hospitalised long COVID group.

ELIGIBILITY CRITERIA

Inclusion criteria

Participants had to meet the following criteria to be eligible for the study:

1. Adults (18 years or older) reporting persistent symptoms lasting at least 8 weeks after initial COVID-19 infection, which interfere with day-to-day activities and who were not hospitalised in the acute infection phase.
2. Individuals recovering at home rather than in a hospital or clinical setting.
3. Access to a compatible mobile device:
    - Android phone (SDK16 or higher) **or** iPhone (iOS version 10 or higher).

Exclusion criteria

Individuals were excluded from the study if they met any of the following criteria:

1. Currently receiving ongoing care for long COVID through primary or secondary healthcare services.
2. Prior diagnosis of a comorbidity with similar symptoms (e.g., ME/CFS).
3. Currently receiving a therapy known to cause symptom exacerbations.
4. Participation in another long COVID-focused intervention at the time of enrolment.
5. Impaired cognitive function that compromises comprehension of study information or the ability to engage with the intervention.
6. Insufficient English language proficiency for effective communication via study messaging.
7. No access to a mobile phone, preventing engagement with the intervention.

STUDY OUTCOMES

Primary and secondary outcomes were designed to meet the domains of the Long COVID core outcome sets (LC-COS). At the time of development, the LC-COS had not been

finalised. However, the initial Delphi survey had been completed. Consequently, the instruments included in the app were selected to reflect each of the major domains that emerged from the Delphi process[10] The development team were also mindful to select instruments that were both valid yet minimised participant burden.

Primary Outcome

The primary outcome of the study is post-exertional malaise (PEM), assessed in terms of its frequency, severity, and duration. This domain captures the impact of activity on symptom exacerbation, including the persistence of symptoms following exertion.

Secondary Outcome(s)

Secondary outcomes include a range of physical, cognitive, and psychosocial health domains relevant to long COVID. These include:

- Overall symptom burden, including self-reported symptom frequency, persistence, and new COVID-19 infections.
- Quality of life, capturing physical, mental, and functional well-being.
- Neurological and cognitive function, including assessments of memory, attention, and nervous system symptoms.
- Respiratory function, specifically breathlessness and its impact on daily activities.
- Mental health, assessing anxiety and depression.
- Pain, measured through self-reported intensity and impact.
- Self-management and self-efficacy, evaluating participants' confidence in managing their condition.

These outcome domains have been selected to reflect the LC-COS core outcome set, ensuring that the study captures key aspects of long COVID that affect daily life.

STUDY PROCEDURES

Recruitment of participants

The following summaries will be presented for all participants screened for entry to the study, by identification or recruitment source and overall. For the purpose of recruitment, the

following summaries will be collected: 1) The number of participants screened, 2) The number of participants recruited, 3) Number and percentage of participants not recruited and the reasons for non-recruitment. Relevant summaries on recruitment, consent and data completeness during follow-up will be presented in a CONSORT flowchart[11]. Reasons for withdrawal at different follow-up times will also be summarised by treatment arm.

Individuals expressing interest to trial information distributed via social media will be contacted via telephone or video conferencing for a briefing which will include screening for inclusion and exclusion criteria, a verbal overview of the study and an opportunity to ask questions. We will provide participants with an information sheet and re-contacted at least 7-days later to provide a further opportunity to ask questions and, if willing, enrol in the trial. Participants will provide written informed consent and then be allocated an enrolment number. Recruitment will be facilitated by our partner organisation, Long COVID Scotland, and involve promotion of the study via online social groups, social media, print media, a study website and meetings with Long COVID Scotland members. We will target people who have not been hospitalised following their COVID-19 infection. We expect to recruit 35 participants per month, and therefore should take 7-months.

Randomisation

Participants will be randomised to one of the two trial arms using 1:1 allocation ratio. Randomisation will be performed by a web-based online randomisation system (Study Randomizer). We will randomise participants remotely and participant blinding is impossible given the nature of the intervention.

Study procedure

The intervention will be a randomised controlled trial (RCT) to determine if adaptive pacing (AP) using activity tracking and just-in-time support messages can improve symptom management of people with long-COVID. The trial will compare the symptom management of people allocated to usual care versus those receiving the intervention. The intervention will be provided via a bespoke support platform incorporating a wearable activity tracker (Fitbit Charge 5, Fitbit, USA), a data processing server, and a cross-platform (iOS and Android) mobile app (PaceMe). Participants will be recruited via online adverts and through long-COVID support groups. Those interested in taking part will undergo a screening interview for

eligibility and were subsequently randomised by a third party (studyrandomizer.com) into intervention or control arms of the trial balanced for gender.

Intervention

Those in the intervention group will be provided with the activity tracker. During enrolment, participants will be guided through the process of turning off all of the notifications and messaging it provided. In addition, they will be helped to download the study support app (PaceMe) and guided through installation and the initial account registration and app onboarding. At enrolment, participants will be allocated time and heart rate (HR) limits of attempting to spend no more than 30 minutes per day above 60% of their age-predicted HRmax. Our separate server will then download the participants' HR for that day in 1-minute intervals from the Fitbit server and calculate the cumulative number of minutes above their HR threshold. The total number of minutes for that day will be displayed in the app, with data downloaded, processed and updated in the app approximately every 3 minutes. Participants will also receive alert notifications when they reached 50, 75, and 100% of their time limits. Alerts include a text notification regarding the percentage of their time limit they have reached, as well as an infographic containing a suggestion for good pacing habits curated from responses from people with ME/CFS who had been using pacing for several years.

The app also allows participants to register when they experienced a bout of PEM. When this occurred, our server will review the 3-days prior to the bout and determine if they exceed their suggested pacing limits in any of those three days. If they had, then there will be no changes to their pacing limits, and participants will receive a notification that we had reviewed their data and that it was likely that they had experienced PEM because they had done too much. If participants had not exceeded their PEM limits, an algorithm will reduce either their HR or time limits, and participants will receive a notification that their bout of PEM might be because their limits were too high and that we had reduced them slightly.

App design and features

To aid data collection the app also includes a series of validated instruments. As a result, in addition to logging a bout of PEM the app includes four 'sections' that participants will be requested to complete monthly: A ‘symptom check in’ to get a view of the month-by-month symptom load of participants. A ‘brain-fog test’ to assess cognitive function using the symbol digit modalities test[13]. The remaining instruments will be split into two groups A and B, with

each group completed at a single point in time. Group A includes the Edinburgh Neurological Survey[14], the modified PEM questionnaire[15], and the SF12 quality of life assessment[16]. Group B includes the MRC breathlessness scale[17], the EQ-5D-5L[18], PHQ4[19], the self-efficacy for long-term conditions[20], and the visual analogue pain scale[21]. Data from each of the instruments will be stored in a GDPR-compliant data server. We also have the data server scan each participant's responses daily and send appropriate notifications to complete one of the four sections each week if responses are missing.

Control participants

Those in the control group will continue with their usual care and follow any support services offered by their general practitioner or other long-COVID support services. Because the study also requires ongoing assessment of symptom load, PEM, and psychometric assessments, the control group will receive a version of the support app in which they could log a bout of PEM, and engage with each of the four sections of the app. They will also receive reminders to complete specific sections, but not receive any activity tracking, information on daily activity, nor any support messaging.

Measurement tools

PRIMARY OUTCOME

De Paul Symptom Questionnaire – Post-exertional malaise (DSQ-PEM)

The baseline date will be considered as the date of baseline data collection. The DSQ-PEM is a 10-item questionnaire. Questions 1-5 are measured on a five-point Likert scale with a 'frequency' domain (0 = none of the time, 1 = a little of the time, 2 = about half the time, 3 = most of the time, and 4 = all of the time) and a 'severity' domain. (0 = symptom not present, 1 = mild, 2 = moderate, 3 = severe, and 4= very severe). Questions 6-8 and 10 are dichotomous yes/no responses, and question 9 asked 'if you feel worse after activities, how long does this last?' with six options: ≤1 h, 2-3 h, 4-10 h, 11-13 h, 14-23 h, or ≥24 h. The DSQ-PEM sum is the sum of questions 1-5 (frequency and severity), expressed out of 100.

SECONDARY OUTCOMES

Patient health questionnaire (PHQ-4)

The baseline date will be considered as the date of baseline data collection. The PHQ-4 is a 4-item questionnaire (Kroenke, Spitzer, Williams, & Lowe, 2009). Questions 1-4 are measured on a four-point Likert scale with 0= not at all, 1= several days, 2=more than half the days,

and 3=nearly every day. The PHQ-4 sum is the scores of each of the 4 items. Scores are rated as normal (0-2), mild (3-5), moderate (6-8) and severe (9-12). Total score ≥3 for first 2 questions suggests anxiety. Total score ≥3 for last 2 questions suggests depression.

Fatigue severity scale (FSS-7)

The baseline date will be considered as the date of baseline data collection. The FSS-7 is a 7-item questionnaire that measures the impact of fatigue (Krupp et al., 1989). Questions 1-7 are measured on a seven-point Likert scale with 1 (strongly disagree), 4 (neither agree nor disagree) and 7 (strongly agree). A visual analogue scale is also included with the scale; respondents are asked to denote the severity of their fatigue over the past 2 weeks by placing a mark on a line extending from “no fatigue” to “fatigue as bad as could be.” Higher scores on the scale are indicative of more severe fatigue.

12-Item Short Form Survey (SF-12)

The baseline date will be considered as the date of baseline data collection. The SF-12 is a 12-item questionnaire that measures self-reported health-related quality of life covering physical (PCS) and mental health (MCS) domains (Ware, Kosinski, & Keller, 1996). Question 1 is measured on a five-point Likert scale with 1=excellent, 2= very good, 3=good, 4=fair and 5=poor. Questions 2-3 are measured on a three-point Likert scale with 1=yes, limited a lot, 2=yes, limited a little, and 3= no, not limited at all. Questions 4-7 are measured are dichotomous yes/no responses. Question 8 is measured on a five-point Likert scale with 1=not at all, 2=a little bit, 3= moderately, 4=quite a bit, and 5=extremely. Questions 9-11 are measured on a six-point Likert scale with 1= all of the time, 2=most of the time, 3=a good bit of the time, 4=some of the time, 5=a little of the time, and 6=none if the time. Question 12 is measured on a five-point Likert scale with 1=all of the time, 2=most of the time, 3=some of the time,4=a little of the time, and 5=none of the time. Scores above 50 indicate a better-than-average health-related quality of life, while scores below 50 suggest below-average health.

EuroQol-5 Dimension (EQ5D)

The baseline date will be considered as the date of baseline data collection. The study uses the EQ-5D-5L version to assess health status and produces a single index value for health status for use in the calculation of quality-adjusted life years to inform health economics evaluation of investigative interventions [10]. The instrument consists of an EQ-5D-5L descriptive system and an EQ-5D-5L visual analogue scale. The descriptive system has 5

dimensions assessing mobility, self-care, usual activity, pain/discomfort, and anxiety. Each of these dimensions has 5 levels of severity which participants are asked to select one of them to best describe their health status 'today': no problems, slight problems, moderate problems, severe problems, and extreme problems. Based on participants' responses from these 5 dimensions, a single index value will be calculated as detailed by Devlin et al [10]. The single index values are on a scale of 0 (full health) to 1 (state equivalent to dead) and health states considered to be worse than dead attain negative values (<0).

General self-efficacy scale (GSE)

The baseline date will be considered as the date of baseline data collection. The GSE is a 10-item questionnaire to assess self-reported self-efficacy (Schwarzer, & Jerusalem, 1995). Questions 1-10 are measured on a four-point Likert scale with 1=not at all true, 2=hardly true, 3=moderately true, and 4=exactly true. The total score is calculated by finding the sum of all items. Total score ranges between 10 and 40 with a higher score indicating more self-efficacy.

Breathlessness (MRC Dypsnoea scale)

The baseline date will be considered as the date of baseline data collection. The MRC Dyspnoea scale is a 5-item questionnaire that assess the degree of baseline functional disability due to dyspnoea (Mahler, & Wellis, 1988). Questions 1-5 are measured on a four-grade scale with 0= I get breathless with strenuous exercise, 1=I get short of breath when hurrying on level ground or walking up a slight hill, 2=On level ground, I walk slower than people of my age because of breathlessness, or I have to stop for breath when walking at my own pace on the level, 3=I stop for breath after walking about 100 yards or after a few minutes on level ground, and 4= I am too breathes to leave the house or I am breathless when dressing/undressing. Total score ranges between 0 and 12 with lower scores indicating worse severity of dyspnoea.

Cognitive function; Smartphone-based symbol digit modalities test (SDMT)

The baseline date will be considered as the date of baseline data collection. The smartphone-based SDMT (Pham et al., 2021), is a smartphone adaptation of the cognitive test, the symbol-digit modalities test (SDMT) examines processing speed and sustained attention by primarily assessing complex visual scanning and tracking. The test compromises of pairing specific numbers with given geometric figures. Responses are given by pressing correct

option on the phone display. Total raw score is calculated as number of correct responses to the total number of all responses given in 90 seconds interval.

Pain Visual Analogue Scale (VAS)

The baseline date will be considered as the date of baseline data collection. The VAS is a validated pain rating scale first developed by Hayes and Patterson (1921), and scores are recorded by dragging a mark on a 10-cm line that represents a continuum between 'no pain' and 'worst pain'. The findings suggested that 100-mm VAS ratings of 0 to 4mm can be considered no pain, 5 to 44 mm, mild pain; 45 to 74 mm, moderate pain; and 75 to 100 mm, severe pain.

The Edinburgh Neurosymptoms Questionnaire (ENS)

The baseline date will be considered as the date of baseline data collection. ENS is a 30-item yes/no survey which include the addition of 241 yes/no sub-questions designed to assess the presence and nature of: blackouts, weakness, hemisensory syndrome, memory problems, tremor, pain, fatigue, globus, multiple medical problems, and operations (Shipston-Sharman et al., 2018).

The Symptom Questionnaire (SQ)

The baseline date will be considered as the date of baseline data collection. The SQ-48 is a 92-item yes/true/no/false questionnaire with brief and simple items state scales of depression, anxiety, anger-hostility, and somatic symptom (Kellner, 1987). Symptom subscales are added together and scored 1 when the answer is YES/TRUE.

STATISTICAL CONSIDERATIONS AND DATA ANALYSIS

Sample size and statistical power

To determine sample size, our primary outcome variable is the DSQ-PEM. Using previous work, a minimum clinically relevant difference can be estimated as a change of 13 points on a 100-point scale [22]. Assuming a standard deviation (SD) of 25 [22], this resulted in a pairwise effect size of d=0.5 (Cohen's f=0.25). We calculated our desired sample size for a two-way mixed-model (within- and between-subjects) analysis of variance (ANOVA). Using the WebPower package in R Studio, and the wp.rmanova function, with two groups, two time points, a medium effect size (f=0.25), assuming sphericity, an alpha of 0.05, desired statistical

power of 0.9, testing for an interaction effect, the total n was 170 (85 per group). Consequently, we aimed to recruit 125 participants per group to allow for 30% drop-out.

Statistical methods

All analyses will be conducted using Jamovi version 2.3.21. Data will be tested for normal distribution and homogeneity of variance to confirm parametric assumptions are met. Data will be presented in text and tables as means and 95% confidence intervals (CI) unless otherwise stated. Because of randomisation, we did not undertake analysis of baseline equivalence, since the null hypothesis must be true and any differences due to chance [11]. Only participants who completed follow-up testing were included in analysis (i.e. per protocol analysis). The effect of the energy management intervention on main and secondary outcomes will be examined using two-way mixed-model ANOVA with condition (intervention or control) as the between-subjects factor and time (pre- and post-intervention) as a within subjects' factor. Alpha level will be reported as exact p values and not described dichotomously as 'significant' or otherwise as recommended by the American Statistical Association [23]. We will express effect sizes from the ANOVA as partial eta-squared (η^2p), with values of 0.01, 0.06, and 0.14 interpreted as small, moderate, and large, respectively [24]. For categorical data, (DSQ-PEM questions 6-10) we will use McNemar's Test for paired samples (pre- to post- intervention), or Chi squared test for between group effects (intervention vs. control).

ETHICAL CONSIDERATIONS

The study will be conducted in full conformance with principles of the "Declaration of Helsinki", Good Clinical Practice (GCP) and within the laws and regulations of the country in which the research is conducted. Following an expression of interest, digital participant information sheets, and study procedures will be shared with potential participants. A further online meeting with one of the research team will be provided to allow for questions and ensure comprehensive of study materials. Participants will give voluntary consent to participate by signing their name within the research app.

All data collected by the application is encrypted and stored on a GDPR compliant server protected by user access rules. For additional security, the app can write data to, but cannot read from, the server; thus, only the research team will have access via a separate authorised

computer. Participants will have the right to terminate the experiment at any point and data was deleted five days following their request to withdrawal their data

OUTCOMES AND SIGNIFICANCE

The significance of this research will be to answer the question as to whether adaptive pacing is effective at reducing long COVID symptom frequency and severity compared to standard care. This could inform symptom management guidelines in a rapidly evolving topic area.

REFERENCES

1. Hayes, L. D., Ingram, J. & Sculthorpe, N. F. More than 100 persistent symptoms of SARS-CoV-2 (long COVID): a scoping review. *Frontiers in Medicine* **8**, (2021).
2. Bayliss, K. *et al.* Overcoming the barriers to the diagnosis and management of chronic fatigue syndrome/ME in primary care: a meta synthesis of qualitative studies. *BMC Fam Pract* **15**, 44 (2014).
3. Deumer, U.-S. *et al.* Myalgic Encephalomyelitis/Chronic Fatigue Syndrome (ME/CFS): An Overview. *Journal of Clinical Medicine* **10**, 4786 (2021).
4. Sanal-Hayes, N. E. M. *et al.* A scoping review of 'Pacing' for management of Myalgic Encephalomyelitis/Chronic Fatigue Syndrome (ME/CFS): lessons learned for the long COVID pandemic. *Journal of Translational Medicine* **21**, 720 (2023).
5. Goudsmit, E. M. & Howes, S. Pacing: A strategy to improve energy management in chronic fatigue syndrome. *Health Psychology Update* **17**, 46 (2008).
6. Sanal-Hayes, N. E. M. *et al.* 'Pacing' for management of myalgic encephalomyelitis/chronic fatigue syndrome (ME/CFS): a systematic review and meta-analysis. *Fatigue: Biomedicine, Health & Behavior* **13**, 36–53 (2025).
7. Hardeman, W., Houghton, J., Lane, K., Jones, A. & Naughton, F. A systematic review of just-in-time adaptive interventions (JITAIs) to promote physical activity. *Int J Behav Nutr Phys Act* **16**, 31 (2019).
8. Perski, O. *et al.* Technology-mediated just-in-time adaptive interventions (JITAIs) to reduce harmful substance use: a systematic review. *Addiction* **117**, 1220–1241 (2022).
9. Cotler, J., Holtzman, C., Dudun, C. & Jason, L. A. A Brief Questionnaire to Assess Post-Exertional Malaise. *Diagnostics* **8**, 66 (2018).

10. Munblat, D., Nicholson, T. & Williamson, P. Personal Communication: Discussion of the initial outcomes of Long-COVID Core Outcome Set (COS). (2021).

11. Moher, D. *et al.* CONSORT 2010 Explanation and Elaboration: updated guidelines for reporting parallel group randomised trials. *BMJ* **340**, c869 (2010).

12. Mahase, E. Covid-19: UK regulator approves lateral flow test for home use despite accuracy concerns. *BMJ* **371**, m4950 (2020).

13. Strober, L. *et al.* Symbol Digit Modalities Test: A valid clinical trial endpoint for measuring cognition in multiple sclerosis. *Mult Scler* **25**, 1781–1790 (2019).

14. Shipston-Sharman, O. *et al.* Screening for functional neurological disorders by questionnaire. *J Psychosom Res* **119**, 65–73 (2019).

15. Cotler, J., Holtzman, C., Dudun, C. & Jason, L. A Brief Questionnaire to Assess Post-Exertional Malaise. *Diagnostics* **8**, 66 (2018).

16. Huo, T., Guo, Y., Shenkman, E. & Muller, K. Assessing the reliability of the short form 12 (SF-12) health survey in adults with mental health conditions: A report from the wellness incentive and navigation (WIN) study. *Health and Quality of Life Outcomes* **16**, (2018).

17. Stenton, C. The MRC breathlessness scale. *Occupational Medicine* **58**, 226–227 (2008).

18. Herdman, M. *et al.* Development and preliminary testing of the new five-level version of EQ-5D (EQ-5D-5L). *Quality of life research* **20**, 1727–1736 (2011).

19. Kroenke, K., Spitzer, R. L., Williams, J. B. & Löwe, B. An ultra-brief screening scale for anxiety and depression: the PHQ–4. *Psychosomatics* **50**, 613–621 (2009).

20. Lorig, K., Chastain, R. L., Ung, E., Shoor, S. & Holman, H. R. Development and evaluation of a scale to measure perceived self-efficacy in people with arthritis. *Arthritis Care Res.* **32**, 37–44 (1989).

21. Delgado, D. A. *et al.* Validation of Digital Visual Analog Scale Pain Scoring With a Traditional Paper-based Visual Analog Scale in Adults. *J Am Acad Orthop Surg Glob Res Rev* **2**, e088 (2018).

22. Jason, L. *et al.* Differentiating Multiple Sclerosis from Myalgic Encephalomyelitis and Chronic Fatigue Syndrome. *Insights Biomed* **2**, 11 (2017).

23. Hurlbert, S. H., Levine, R. A. & Utts, J. Coup de Grâce for a Tough Old Bull: "Statistically Significant" Expires. *The American Statistician* **73**, 352–357 (2019).

24. Lakens, D. Calculating and reporting effect sizes to facilitate cumulative science: a practical primer for t-tests and ANOVAs. *Front Psychol* **4**, (2013).
