# Supplementary material for: A digital platform with activity tracking for energy management support in long COVID: a randomised controlled trial
Source: Nat Commun. 2026 Feb 2;17:945. doi: 10.1038/s41467-025-64831-y (PMC12864992; doi:10.1038/s41467-025-64831-y)
Supplement: Supplementary file 3 — Reporting Summary [file 41467_2025_64831_MOESM3_ESM.pdf]

nature portfolio

Corresponding author(s): Lawrence Hayes

Last updated by author(s): Sep 3, 2025

Reporting Summary

Nature Portfolio wishes to improve the reproducibility of the work that we publish. This form provides structure for consistency and transparency in reporting. For further information on Nature Portfolio policies, see our Editorial Policies and the Editorial Policy Checklist.

Statistics

For all statistical analyses, confirm that the following items are present in the figure legend, table legend, main text, or Methods section.

n/a	Confirmed	
☐	☒	The exact sample size (*n*) for each experimental group/condition, given as a discrete number and unit of measurement
☐	☒	A statement on whether measurements were taken from distinct samples or whether the same sample was measured repeatedly
☐	☒	The statistical test(s) used AND whether they are one- or two-sided<br>*Only common tests should be described solely by name; describe more complex techniques in the Methods section.*
☐	☒	A description of all covariates tested
☐	☒	A description of any assumptions or corrections, such as tests of normality and adjustment for multiple comparisons
☐	☒	A full description of the statistical parameters including central tendency (e.g. means) or other basic estimates (e.g. regression coefficient) AND variation (e.g. standard deviation) or associated estimates of uncertainty (e.g. confidence intervals)
☐	☒	For null hypothesis testing, the test statistic (e.g. *F*, *t*, *r*) with confidence intervals, effect sizes, degrees of freedom and *P* value noted<br>*Give P values as exact values whenever suitable.*
☒	☐	For Bayesian analysis, information on the choice of priors and Markov chain Monte Carlo settings
☒	☐	For hierarchical and complex designs, identification of the appropriate level for tests and full reporting of outcomes
☐	☒	Estimates of effect sizes (e.g. Cohen's *d*, Pearson's *r*), indicating how they were calculated

Our web collection on statistics for biologists contains articles on many of the points above.

Software and code

Policy information about availability of computer code

Data collection	Data collection were conducted using validated questionnaires. The primary outcome was the DePaul Symptom Questionnaire - PEM (DSQ-PEM).<br><br>Secondary outcomes were 12-Item Short Form Health Survey (SF-12), EuroQol 5-Dimension Health Questionnaire (EQ5D), Pain Visual Analogue Scale (VAS), Patient Health Questionnaire-4 (PHQ4), Fatigue Severity Scale (7-item version; FSS-7), Medical Research Council Breathlessness Questionnaire (MRC BQ), Symbol Digit Modalities Test (SDMT) total correct (out of 60), total time for correct answers only, average time per correct answer.
Data analysis	To determine sample size, our primary outcome variable was the DSQ-PEM. Using previous work, a minimum clinically relevant difference can be estimated as a change of 13 points on a 100-point scale 37. Assuming a standard deviation (SD) of 25 37, this resulted in an effect size of f=0.25. We calculated our desired sample size for a two-way mixed-model (within- and between-subjects) analysis of variance (ANOVA). Using the WebPower package in R Studio (version 2024.04.2+764), and the wp.rmanova function, with two groups, two time points, f=0.25, assuming sphericity, α=0.05, 1-β=0.9, testing for an interaction effect, the total n was 170 (85 per group). Consequently, we aimed to recruit 125 participants per group to allow for 30% drop-out.<br>All analyses were conducted using Jamovi version 2.3.21. Data were tested for normal distribution and homogeneity of variance to confirm parametric assumptions were met. Data are presented in text and tables as means and 95% confidence intervals (CI) unless otherwise stated. Because of randomisation, we did not undertake analysis of baseline equivalence, since the null hypothesis must be true and any differences due to chance 38. Only participants who completed follow-up testing were included in the primary analysis (i.e. per protocol analysis). The effect of the intervention on primary and secondary outcomes was examined using two-way mixed-model ANOVAs with condition (intervention or control) as the between-subjects factor and time (pre- and post-intervention) as a within subjects factor. To confirm our assumptions were robust, we conducted sensitivity analysis in the form of intention to treat analysis for all participants who were randomised.

In the case of missing data, mixed effects models were used to account for the repeated measures structure of the data and to provide robust handling of missing data. Unlike non-parametric methods, which require complete case analysis and often assume homogeneity of variance across groups, mixed-effects models offer greater flexibility by allowing the inclusion of data under the assumption that data are missing at random. The linear mixed-effects model was specified for DSQ-PEM sum score (primary outcome variable), and DSQ-PEM questions 1-5, with time (seven monthly time points) and group (intervention and control) as fixed effects and subject ID included as a random intercept to account for individual variability. No outcome distributions deviated from normality, so no data transformations were applied. In the case of main effects, location of differences was further investigated using the Tukey post-hoc test. Alpha level is reported as exact p values and not described dichotomously as 'significant' or otherwise as recommended by the American Statistical Association 39. We expressed effect sizes from the ANOVA as partial eta-squared (η^2p), with values of 0.01, 0.06, and 0.14 interpreted as small, moderate, and large, respectively 40. For categorical data, (DSQ-PEM questions 6-10, and DSQ-PEM screening as positive or negative) we used McNemar's Test for paired samples (pre- to post- intervention), or Chi squared test for between group effects (intervention vs. control).

For manuscripts utilizing custom algorithms or software that are central to the research but not yet described in published literature, software must be made available to editors and reviewers. We strongly encourage code deposition in a community repository (e.g. GitHub). See the Nature Portfolio guidelines for submitting code & software for further information.

Data

Policy information about availability of data

All manuscripts must include a data availability statement. This statement should provide the following information, where applicable:

- Accession codes, unique identifiers, or web links for publicly available datasets
- A description of any restrictions on data availability
- For clinical datasets or third party data, please ensure that the statement adheres to our policy

Data collected for this study, including individual anonymised participant data is available to others via Figshare: 10.6084/m9.figshare.30053614
The study protocol is available from https://www.isrctn.com/ISRCTN16033549.

Research involving human participants, their data, or biological material

Policy information about studies with human participants or human data. See also policy information about sex, gender (identity/presentation), and sexual orientation and race, ethnicity and racism.

Reporting on sex and gender	Participants were requested to self-report their 'gender' within our study. We conducted no gender comparisons within our study.<br><br>However, to confirm our results were relevant for both genders, we disaggregated the results. In females only, repeated measures ANOVA revealed an effect of time (p=0.021, η^2p=0.041; small), but not group (p=0.825, η^2p=0.000; trivial), or interaction effect (p=0.358, η^2p=0.007; trivial) for the primary outcome variable (sum of the DSQ-PEM). The intervention group value was 50 (95% CI 45-55) at baseline and 43 (95% CI 38-49) post-intervention. The control group value was 49 (95% CI 43-54) at baseline and 46 (95% CI 40-52) at follow-up. In males only, repeated measures ANOVA revealed no effect of time (p=0.275, η^2p=0.038; small), or group (p=0.057, η^2p=0.112; moderate), but there was an interaction effect (p=0.045, η^2p=0.123; moderate) for the primary outcome variable. The intervention group value was 43 (95% CI 33-53) at baseline and 56 (95% CI 46-65) post-intervention. The control group value was 41 (95% CI 30-52) at baseline and 37 (95% CI 27-46) at follow-up.
Reporting on race, ethnicity, or other socially relevant groupings	We did not collect these information.
Population characteristics	We only have age data. This was used to provide the heart rate limit for the intervention. Again, given the newness of long COVID and digital energy management, we have no biological plausibility to test this hypothesis.
Recruitment	Individuals expressed interest to trial information on social media by sharing their email address, or contacting our PPI partner (Long COVID Scotland), or contacting us directly via email. We emailed interested parties an information sheet and asked them to review the material and respond after 48 hours. If there was no response, we followed up with a reminder email asking if they were still interested in participating. Those who confirmed their interest were scheduled for an online meeting or a phone call, during which we reviewed inclusion and exclusion criteria, provided a brief overview of the study, and offered time for questions about the trial. Participants chose whether they wished to enrol in the study, and we obtained their verbal consent to proceed with randomisation during that meeting. Recruitment was assisted by our partner organisation, Long COVID Scotland, and involved promotion of the trial via social media groups, print media, a study website, and meetings with Long COVID Scotland members. The trial targeted people who had not been hospitalised following their COVID-19 infection, therefore there was no data linkage or recruitment via primary or secondary care.
Ethics oversight	The study was approved by the University of the West of Scotland Institutional Ethics Board (approval number 16638)

Note that full information on the approval of the study protocol must also be provided in the manuscript.

Field-specific reporting

Please select the one below that is the best fit for your research. If you are not sure, read the appropriate sections before making your selection.

☒ Life sciences ☐ Behavioural & social sciences ☐ Ecological, evolutionary & environmental sciences

For a reference copy of the document with all sections, see nature.com/documents/nr-reporting-summary-flat.pdf

Life sciences study design

All studies must disclose on these points even when the disclosure is negative.

Sample size	To determine sample size, our primary outcome variable was the DSQ-PEM. Using previous work, a minimum clinically relevant difference can be estimated as a change of 13 points on a 100-point scale 18. Assuming a standard deviation (SD) of 25 18, this resulted in a pairwise effect size of d=0.5 (Cohen's f=0.25). We calculated our desired sample size for a two-way mixed-model (within- and between-subjects) analysis of variance (ANOVA). Using the WebPower package in R Studio, and the wp.rmanova function, with two groups, two time points, a medium effect size (f=0.25), assuming sphericity, an alpha of 0.05, desired statistical power of 0.9, testing for an interaction effect, the total n was 170 (85 per group). Consequently, we aimed to recruit 125 participants per group to allow for 30% drop-out. A post-hoc power calculation resulted in observed power of 0.88 with our sample size of 161, an effect size of f=0.25, and an alpha level of 0.05.
Data exclusions	Only participants who completed follow-up testing were included in the primary analysis (i.e. per protocol analysis).
Replication	A range of measures were implemented to ensure that our experimental findings were reproducible:<br>Detailed Protocol Documentation: Every step of the experimental procedure was rigorously documented. This comprehensive record allows both internal and external researchers to replicate the exact conditions under which the original data were collected.<br>Internal Replication:Multiple independent researchers within our laboratory repeated the data analysis using different software, and came to the same result. These replications were performed blindly to minimize bias.<br>Pre-Registered Methodology:The experimental design, along with the statistical analysis plan, was pre-registered. This step ensured that the procedures were strictly followed and that any deviations or post hoc adjustments were transparently reported.<br><br>Thus, we believe we have taken every step to ensure repeatability, without re-running the whole study.
Randomization	A secure third-party service (studyrandomizer.com) was used to randomise 250 participants 1:1 into two evenly distributed study arms (intervention or control) with each arm consisting of 125 participants (50% of the total sample; equal allocation). Randomisation into the respective arms was conducted using a permuted block algorithm, with a fixed block size of 125 participants. Participants randomised to the intervention group received usual care with just-in-time messaging support, and the control group received usual care only (Figure 1). NS-H generated the sequence, enrolled participants, and assigned them to the trial groups. NS-H was the senior post-doctoral trial manager for the rest of the trial.
Blinding	Blinding was impossible due to the requirements of the experimental group.

Reporting for specific materials, systems and methods

We require information from authors about some types of materials, experimental systems and methods used in many studies. Here, indicate whether each material, system or method listed is relevant to your study. If you are not sure if a list item applies to your research, read the appropriate section before selecting a response.

Materials & experimental systems

n/a	Involved in the study	
☒	☐	Antibodies
☒	☐	Eukaryotic cell lines
☒	☐	Palaeontology and archaeology
☒	☐	Animals and other organisms
☐	☒	Clinical data
☒	☐	Dual use research of concern
☒	☐	Plants

Methods

n/a	Involved in the study	
☒	☐	ChIP-seq
☒	☐	Flow cytometry
☒	☐	MRI-based neuroimaging

Clinical data

Policy information about clinical studies

All manuscripts should comply with the ICMJE guidelines for publication of clinical research and a completed CONSORT checklist must be included with all submissions.

Clinical trial registration	ISRCTN16033549

Study protocol	The full protocol will be available via ISRCTN16033549
Data collection	Data were collected remotely. The trial ran between January 2022 and September 2023; recruitment ran between November 2021 and February 2023.
Outcomes	Primary outcome:<br>Post-exertional malaise using the DSQ-PEM at baseline and 6 months.<br><br>Secondary outcomes:<br>1. PEM trend using PEM questionnaire assessed monthly<br>2. Depression trend using PHQ-9 monthly<br>3. Depression change using PHq-9 assessed at baseline and 6 months<br>4. Fatigue trend using FSS monthly<br>5. Fatigue change using FSS at baseline and 6 months<br>6. Quality of life trend using SF12 monthly<br>7. Quality of life change using SF12 at baseline and 6 months<br>8. Self-efficacy trend using Self Efficacy Scale monthly<br>9. Cognitive function using a variation of the symbol digit modalities test monthly<br>10. Cognitive function using a variation of the symbol digit modalities test at baseline and 6 months.<br>11. Pain using Pain VAS assessed at baseline and 6 months<br>12. Pain trend using Pain VAS assessed monthly between months 0-6<br><br>The effect of the intervention on primary and secondary outcomes was examined using two-way mixed-model ANOVAs with condition (intervention or control) as the between-subjects factor and time (pre- and post-intervention) as a within subjects factor.

Plants

Seed stocks	*Report on the source of all seed stocks or other plant material used. If applicable, state the seed stock centre and catalogue number. If plant specimens were collected from the field, describe the collection location, date and sampling procedures.*
Novel plant genotypes	*Describe the methods by which all novel plant genotypes were produced. This includes those generated by transgenic approaches, gene editing, chemical/radiation-based mutagenesis and hybridization. For transgenic lines, describe the transformation method, the number of independent lines analyzed and the generation upon which experiments were performed. For gene-edited lines, describe the editor used, the endogenous sequence targeted for editing, the targeting guide RNA sequence (if applicable) and how the editor was applied.*
Authentication	*Describe any authentication procedures for each seed stock used or novel genotype generated. Describe any experiments used to assess the effect of a mutation and, where applicable, how potential secondary effects (e.g. second site T-DNA insertions, mosiacism, off-target gene editing) were examined.*
